# Impact of chronic endometritis on endometrial receptivity analysis results and pregnancy outcomes

**DOI:** 10.1002/iid3.354

**Published:** 2020-09-23

**Authors:** Keiji Kuroda, Takashi Horikawa, Azusa Moriyama, Kazuki Nakao, Hiroyasu Juen, Satoru Takamizawa, Yuko Ojiro, Koji Nakagawa, Rikikazu Sugiyama

**Affiliations:** ^1^ Center for Reproductive Medicine and Implantation Research Sugiyama Clinic Shinjuku Tokyo Japan; ^2^ Department of Obstetrics and Gynecology Juntendo University Faculty of Medicine Tokyo Japan

**Keywords:** chronic endometritis, endometrial receptivity analysis, infertility, personalized embryo transfer, reproductive failure, window of implantation

## Abstract

**Background:**

The aim of this study is to evaluate the relationship between chronic endometritis (CE) and a personalized window of implantation (WOI), identified by results of endometrial receptivity analysis (ERA), and pregnancy outcomes following embryo transfer (ET) based on the ERA outcomes.

**Methods:**

The single‐center, cross‐sectional study was designed. The study population consisted of 101 infertile women who underwent endometrial sampling between June 2018 and February 2020. We recruited 88 patients who underwent ERA testing and immunohistochemistry of the plasma cell marker CD138 to diagnose CE within 3 months of testing. Subjects were divided into three groups as follows: 33 without CE (non‐CE group); 19 with untreated CE at ERA testing (CE group); and 36 successfully treated for CE before ERA testing (cured‐CE group). CE diagnosis was defined as ≥5 CD138‐positive plasma cells per 10 random stromal areas at ×400 magnification.

**Results:**

In non‐CE, CE, and cured‐CE groups, the numbers of CD138‐positive cells were 0.7 ± 1.0, 28.5 ± 30.4, and 1.3 ± 1.3, respectively (*p* < .001). The rates of “receptive” endometrium in non‐CE and cured‐CE groups were 57.6% (19 women) and 50.0% (18 women), respectively; however, in the CE group, this rate was significantly lower than the other two groups (*p* = .009) at only 15.8% (3 women). After CE were treated prior or posterior to the ERA test in cured‐CE or CE groups, the clinical pregnancy rates at the first ET in non‐CE, CE, and cured‐CE groups were 77.8% (21/27 cycles), 22.2% (4/18 cycles), and 51.7% (15/29 cycles), respectively (*p* < 0.001).

**Conclusion:**

CE had detrimental effects on the individual WOI, leading to embryo–endometrial asynchrony; therefore, diagnosis and treatment of CE should be done before ERA testing.

## Graphical abstract

To confirm an impact of chronic endometritis (CE) on the results of endometrial receptivity analysis (ERA) testing, subjects were divided into three groups as follows: 33 without CE (non‐CE group); 19 with untreated CE at ERA testing (CE group); and 36 successfully treated for CE before ERA testing (cured‐CE group). The rates of “receptive” endometrium in non‐CE and cured‐CE groups were 57.6% (19 women) and 50.0% (18 women), respectively; however, in the CE group, this rate was significantly lower than the other two groups (*p* = .009) at only 15.8% (3 women). CE had detrimental effects on the ERA results.

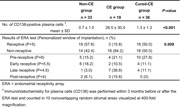

## 1 INTRODUCTION

In humans, synchronization of a developmentally competent embryo and optimal decidualization of endometrium is indispensable for successful pregnancy.^1^ The process of transforming endometrial stromal cells into decidual cells plays an important role in dynamic functional changes, including immunomodulation for embryo receptivity, acquirement of oxidative stress defenses, remodeling of specialized vascularity, and control of trophoblast invasion.^1^ Decidual transformation of the endometrium triggers an acute inflammatory reaction that is followed by an anti‐inflammatory response. This inflammatory secretome switch in decidualization harmonizes with the period of embryo receptivity known as the window of implantation (WOI). Recent evidence has shown that decidualized endometrial cells serve as biosensors of embryo quality upon implantation.^2^, ^3^, ^4^, ^5^ Therefore, impaired decidualization of the endometrium is associated with implantation failure and pregnancy complications, such as pregnancy loss and perturbation of placental formation.^3^, ^6^, ^7^, ^8^

Noyes et al.^9^, ^10^ were the first to define the complex transition in the human endometrium during the menstrual cycle using endometrial dating of immunostained tissues. However, subsequent studies revealed that it is difficult to accurately determine a WOI via histological endometrial dating, and that the traditional criteria involve high interobserver variability.^11^, ^12^ In recent years, endometrial receptivity‐associated genes in humans have been identified using molecular comprehensive analysis.^4^, ^13^, ^14^ Díaz‐Gimeno et al.^13^, ^15^ identified 238 candidate endometrial receptivity‐related genes and their gene expression profiles using microarray technology that compares human pre‐decidualized and decidualized endometrium. Now known as the endometrial receptivity analysis (ERA), these authors describe ERA as a novel tool for objectively identifying an optimal WOI based on analysis of the expression of these genes by a computational predictor.^13^ The benefits and efficacy of ERA testing and embryo transfer (ET) at the time of optimal WOI has been clarified; however, the therapeutic effect of ERA testing for the patients with a history of repeated implantation failure (RIF) is still questioned.^16^, ^17^, ^18^, ^19^

Menstruation has a significant anti‐pathogenic role, in which menstrual blood delivers pathogenic microorganisms from the uterine cavity to the vagina.^20^ Chronic endometritis (CE) is a continuous inflammatory condition of local endometrium that persists across the different phases of the menstrual cycle. Approximately 30%–60% of infertile women with a history of RIF have CE.^21^, ^22^, ^23^, ^24^, ^25^ Recovery from CE can lead to dramatic improvements in pregnancy outcomes in IVF treatment.^25^, ^26^, ^27^ CE is caused by a wide variety of microorganisms, is basically asymptomatic, and is undetectable by common testing methods for infertility.^23^ The gold standard of diagnosis for CE is histopathological identification using immunostaining of the plasma cell marker CD138.^28^ Recent in vitro studies have clarified that CE is associated with decreased expression of embryo receptivity‐associated genes and decidual markers.^29^, ^30^ Therefore, CE may adversely affect decidualization of endometrium and formation of an optimal WOI; however, the clinical impact of CE on the ERA test has not been reported. This study aimed to evaluate the relationship between CE and individual WOI identified by the ERA, as well as pregnancy outcomes in ET cycles based on ERA outcomes.

## 2 MATERIALS AND METHODS

### 2.1 Study design

**Figure 1:**
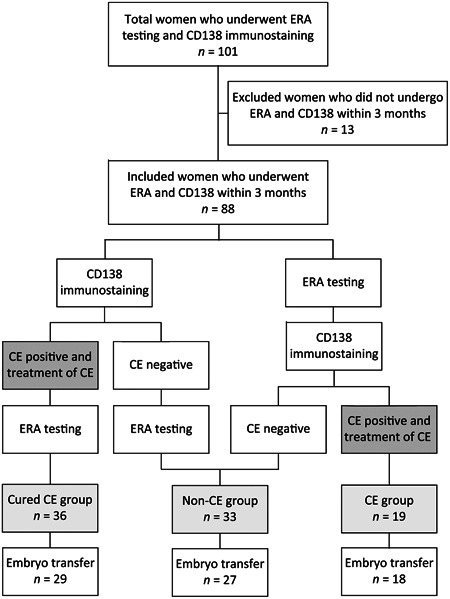
Flowchart of patient selection. Of consecutive 101 infertile women who underwent endometrial biopsy for ERA testing and CD138 immunostaining between June 2018 and February 2020, 88 women who had undergone both tests within 3 months were recruited. As shown in figure, we divided the patients into three groups as follows: women without CE (non‐CE group, *n* = 33); women with untreated CE at the time of ERA testing (CE group, *n* = 19); and women successfully treated for CE before ERA testing (cured‐CE group, *n* = 36). As for pregnancy outcomes, we focused on 74 women who underwent embryo transfer at least once in our clinic. CE, chronic endometritis; ERA, endometrial receptivity analysis

This was a single‐center, cross‐sectional study. All endometrial biopsies for immunohistochemistry (IHC) of CD138 and the ERA test were performed in the Sugiyama Clinic Shinjuku in Tokyo, Japan, between June 2018 and February 2020 (Figure 1). Of consecutive 101 women with infertility who underwent the ERA test, we recruited 88 patients who had undergone both the ERA test and IHC for CD138 within 3 months. None of the patients had any intrauterine pathology via hysteroscopy. As shown in Figure 1, we divided the patients into three groups as follows: women without CE (non‐CE group, *n* = 33); women with untreated CE at the time of ERA testing (CE group, *n* = 19); and women successfully treated for CE before ERA testing (cured‐CE group, *n* = 36). As for pregnancy outcomes, ET was performed after CE were treated prior or posterior to the ERA test in the cured‐CE or CE group. We focused on 74 women, including 27, 18, and 29 women in the non‐CE, CE, and cured‐CE groups, respectively, who underwent ET at least once in our clinic, of whom 63 underwent two or more ETs or conceived at the first ET to derive the cumulative ongoing pregnancy rate. The study protocol was approved by the Ethics Committee of Sugiyama Clinic, Tokyo, Japan (No.18‐004).

### 2.2 Endometrial biopsy for CD138 immunohistochemistry and ERA

To conduct IHC for CD138, endometrial samples were obtained from the patients using endometrial suction curette (Pipet Curet; Fuji Medical Corporation) at the day of ovulation or later, within 3 months before or after ERA. The tissue samples were divided into two; one was fixed in 10% formaldehyde for immunostaining using anti‐CD138 antibody (M7228; Dako) and the other was kept in the tube for intrauterine bacterial culture with drug sensitivity. Both samples were sent to BML, Inc. The pathologists counted CD138‐positive plasma cells in 10 nonoverlapping random stromal areas visualized at 400‐fold magnification (BML), from which we diagnosed CE as the presence of ≥5 CD138‐positive cells.

For the ERA test, the preparation of the endometrium was accomplished via a hormone replacement cycle. From Day 3 of the menstrual cycle, a transdermal estradiol patch (Estrana Tape 0.72 mg; Hisamitsu Pharmaceutical) and conjugated estrogen tablets (Premarin 0.625 mg; Wyeth) were administered. At Day 13, ≥7 mm of endometrial thickness was confirmed, and the patients were started on 30 mg dydrogesterone tablets (Duphastone 5 mg; Abbott) three times daily and 90 mg vaginal progesterone gel (OneCrinone 90 mg; Merck) once daily from day 14 for 5 days. Endometrial tissue was biopsied using Pipet Curet 5 days after initiation of progesterone administration (P + 5). The biopsied endometrial tissue was placed in a cryotube, shaken a few times, and stored at 4°C for 4 h or more according to the manufacturer's protocol. It was then transferred at room temperature to Igenomix Japan, where the ERA analysis was performed. The expression of the endometrial receptivity‐associated genes was analyzed using a customized microarray, as described previously.^13^, ^15^ The ERA demonstrates each patient's individual WOI as “receptive” or “non‐receptive.” Non‐receptive is further classified as pre‐receptive, early receptive, late receptive and postreceptive, indicating that state at the time of endometrial biopsy at 24 h earlier, 12 h earlier, 12 h later, and 24 h later than the personalized WOI, respectively. Therefore, in a subsequent cycle, we performed ET at P + 6, P + 5.5, P + 5, P + 4.5, or P + 4 days in the patients who were at the prereceptive, early receptive, receptive, late receptive, or postreceptive stage, respectively. All ETs were performed at the time designated by the ERA test, using vitrified blastocysts at developmental Stage 4 or more in the Gardner classification.^31^

### 2.3 Treatment of chronic endometritis

The treatment protocol for CE was administration of oral doxycycline (Vibramycin® tablets; Pfizer Japan Inc.), 100 mg twice a day for 2 weeks as the first‐line according to previous reports.^21^, ^32^ We performed endometrial biopsy for IHC of CD138 and intrauterine bacterial culture with drug sensitivity during the luteal phase more than 1 week after the day of completion of antibiotic administration. If the patient had not recovered from CE and the culture test detected specific bacteria other than *Lactobacillus spp*. or *Bifidobacterium spp*., we selected appropriate antibiotics according to the results of drug sensitivity and administered bacterium‐sensitive antibiotics for 2 weeks as the second‐line. When a specific bacterium could not be determined, a combination of amoxicillin (Sawacillin tablets, 250 mg; LTL Pharma Co., Ltd.), azithromycin (Azithromycin tablets, 250 mg; Fuji Pharma Co., Ltd.), metronidazole (Flagyl, 250 mg; Shionogi & Co., Ltd.), and antibiotic‐resistant lactic acid bacteria (Biofermin‐R® tablets, 6.0 mg; Biofermin pharmaceutical Co., Ltd.), were administered twice a day for 2 weeks. If CD138‐positive plasma cells had not disappeared after 2–3 cycles of antibiotic therapy and specific bacteria were not detected, uterine endometrium was shed artificially by dilatation and curettage (D&C).

### 2.4 Ovarian stimulation and IVF‐ET

Procedures for ovarian stimulation, oocyte retrieval, and IVF‐ET were described previously.^33^, ^34^ Briefly, a mild stimulation protocol comprising a clomiphene citrate or letrozole in combination with recombinant follicle‐stimulating hormone (rFSH) or human menopausal gonadotropin (hMG) cycle was used as follows: patients were administered 2.5 mg letrozole (Letrozole tablets, 2.5 mg; Kobayashi Kako Co., Ltd) once daily for 5 days, or 50–100 mg clomiphene citrate (Clomid; Fuji Pharma) once or twice daily for 5–10 days from Day 3 of the menstruation cycle, and injection of 150–300 IU rFSH (Gonal‐f; Merck) or hMG (HMG Ferring; Ferring Pharmaceuticals) on Days 3, 4, 6, and 8. On menstrual day 10, when dominant follicles were developed to ≥17 mm, either 250 µg recombinant human chorionic gonadotropin (hCG) (Ovidrel; Merck) injection or 600 µg nasal buserelin acetate spray (Buserecur; Fuji Pharma Co.) was administered for ovulation induction and oocyte maturation. At 35 h after ovulation induction, oocyte retrieval was performed transvaginally. Conventional IVF or intracytoplasmic sperm injection (ICSI) was selected, depending on sperm findings and fertilization rates in previous IVF treatment cycles. All embryos were cryopreserved at blastocyst developmental stage ≥4 in the Gardner classification using the vitrification method.^35^ Morphologically good embryos were diagnosed as 5‐ or 6‐day blastocysts after conventional IVF or ICSI, except for grade C in both the inner cell mass and the trophectoderm of the Gardner classification. In the vitrified‐warmed ET cycle, the endometrium was prepared for ET via a hormone replacement cycle in the same way as the ERA test. Blastocysts were placed into the maternal uterus using a soft catheter (Kitazato ET Catheter, 6Fr; Kitazato Corporation) guided by transvaginal ultrasound, based on the results of ERA, as a personalized ET. Serum hCG level was examined at 9 days after ET, and clinical pregnancy was diagnosed when a gestational sac was detected using transvaginal ultrasound at 16 days after ET (5 weeks of gestation). A miscarriage was defined as a case of loss in clinical pregnancy during 5 to 12 weeks of gestation. Ongoing pregnancy was defined as maintenance of pregnancy at 12 weeks or later of gestation.

### 2.5 Statistical analysis

Statistical analysis was performed using the GraphPad Prism 5 (GraphPad Software Inc.). Differences among continuous variables of three groups were analyzed using the Kruskal–Wallis test or Fischer's exact test, as appropriate. The level of significance was defined as a *p* < .05.

## 3 RESULTS

### 3.1 CE effects on ERA results

The characteristics of the patients are summarized in Table 1. The patient age at the ERA, duration of infertility, history of pregnancy, causes of infertility, serum anti‐Müllerian hormone level, and number of previous ET cycles were not significantly different in the three groups. Half of the patients had a history of RIF which was diagnosed implantation failure after three or more ET cycles. In the non‐CE, CE, and cured‐CE groups, the number of CD138‐positive plasma cells were 0.7 ± 1.0, 28.5 ± 30.4, and 1.3 ± 1.3 per 10 nonoverlapping random stromal areas within 3 months before or after the ERA (Table 2), and the difference among three groups was significant (*p* < .001). The ERA results revealed proportions of endometria rated as receptive to non‐receptive in the non‐CE and cured‐CE groups to be 57.6% (19/33 women) and 50.0% (18/36 women), respectively; however, in the CE group, only 15.8% (3/19 women) were receptive (*p* < .001 compared with the other two groups). Although most of the patients with CE had non‐receptive endometria, the rates of receptive endometria in the non‐CE and cured‐CE groups were essentially the same. This result suggests that appropriate treatment of CE may allow recovery from an impaired WOI.

**Table 1:** Characteristics of infertile women who underwent ERA test

	**Non‐CE group**	**CE group**	**Cured‐CE group**	***p* Value**
	***n* = 33**	***n* = 19**	***n* = 39**	
Age at ERA test, years, mean ± *SD* (range)	36.9 ± 3.9	38.3 ± 3.7	38.4 ± 4.0	.334
	(29–44)	(33–45)	(29–46)	
Duration of infertility, years, mean ± *SD*	3.1 ± 2.3	2.7 ± 1.5	3.6 ± 2.5	.713
History of pregnancy, median (range)				
Gravida	0 (0–3)	0 (0–4)	0 (0–4)	.493
Para	0 (0–1)	0 (0)	0 (0–3)	.465
Causes for infertility, *n* (%)				
Tubal factors	1 (3.0)	2 (10.5)	0 (0)	.110
Endometriosis	5 (15.1)	2 (10.5)	2 (5.6)	.433
Ovarian factors	2 (6.0)	0 (0)	1 (2.8)	.605
Male factors	4 (12.1)	2 (10.5)	5 (13.9)	1.000
Unexplained	18 (54.5)	14 (73.7)	22 (61.1)	.425
Serum AMH level, ng/ml, mean ± *SD*	4.2 ± 3.4	3.3 ± 2.6	2.9 ± 2.7	.089
No. of previous embryo transfer	3.4 ± 4.0	2.1 ± 2.3	3.4 ± 3.2	.484
Rate of RIF^a^	17 (51.5)	9 (47.4)	20 (55.6)	.857

Abbreviations: AMH, anti‐Müllerian hormone; CE, chronic endometritis; ERA, endometrial receptivity array; RIF, repeated implantation failure.

^a^ RIF was diagnosed as history of implantation failure after three or more embryo transfer cycles.

**Table 2:** Outcomes of immunohistochemistry for CD138‐positive plasma cells and ERA test

	Non‐CE group	CE group	Cured‐CE group	*p* Value
	*n* = 33	*n* = 19	*n* = 36	
No. of CD138‐positive plasma cells^a^, mean ± *SD*	0.7 ± 1.0	28.5 ± 30.4	1.3 ± 1.3	**<.001**
Results of ERA test (personalized window of implantation), *n* (%)				
Receptive (P + 5)	19 (57.6)	3 (15.8)	18 (50.0)	**.009**
Non‐receptive	14 (42.4)	16 (84.2)	18 (50.0)	
Pre‐receptive (P + 6)	5 (15.2)	4 (21.1)	10 (27.8)	
Early receptive (P + 5.5)	6 (18.2)	2 (10.5)	4 (11.1)	
Late receptive (P + 4.5)	1 (3.0)	7 (36.8)	4 (11.1)	
Post‐receptive (P + 4)	2 (6.1)	3 (15.8)	0 (0)	

Abbreviation: CE, chronic endometritis; ERA, endometrial receptivity array.

^a^ Immunohistochemistry for plasma cells (CD138) was performed within 3 months before or after the ERA test and counted in 10 nonoverlapping random stromal areas visualized at 400‐fold magnification.

### 3.2 ERA results before and after treatment of CE

Two patients out of three in the CE group who underwent the ERA twice (once before and once after CE treatment) showed ERA‐receptive endometria after antibiotic therapy. One of these cases is described below and the treatment timeline is depicted in Figure S1.

Case: The patient was a 42‐year‐old woman with a history of RIF after four ET cycles in a previous hospital who underwent endometrial biopsy for the ERA test IHC of CD138, and intrauterine bacterial culture to test for RIF at our hospital. The first biopsy showed post‐receptive endometria with 90 ± 3 h as the recommended timing of ET, 98 CD138‐positive cells in 10 nonoverlapping random stromal areas, and no specific bacteria by intrauterine bacterial culture. The patient was diagnosed with CE and administered doxycycline, 100 mg twice daily for 2 weeks. The second biopsy, which was obtained one week later after antibiotic therapy, showed 30 CD138‐positive cells and no specific detectable bacteria; thus, a combination of amoxicillin, azithromycin, metronidazole, and antibiotic‐resistant lactic acid bacteria, twice daily for 2 weeks, was further administered as our treatment protocol. At the third biopsy, CE was finally considered cured based on results of CD138‐positivity (1 cell) and identification of *Candida glabrata* on intrauterine bacterial culture. As a precaution, fluconazole (Diflucan; Pfizer Japan Inc.) 100 mg once daily for 2 weeks was administered. The patient underwent two ET cycles using two morphologically good blastocysts at 90 h after starting progesterone in a hormonal replacement cycle; however, serum hCG levels were not detected at 9 days after ET. Considering the improved intrauterine environment and endometrial function following treatment for CE, we conducted a fourth biopsy for the ERA. It demonstrated an optimal WOI, with receptive endometria and 126 ± 3 h as the recommended timing for ET. Finally, ET using blastocyst (Grade 5AB) at 126 h after starting progesterone resulted in successful pregnancy.

### 3.3 Pregnancy outcomes after personalized embryo transfer based on ERA results

When CE is recognized, we treat all cases before ET as described for the patient above. Recovery ratios in the CE and cured‐CE groups are shown in Table 3. All patients recovered from CE within three cycles of treatment with antibiotics and/or D&C. Pregnancy outcomes after the ERA and personalized ET in the non‐CE, CE, and cured‐CE groups were 77.8% (21/27 cycles), 22.2% (4/18 cycles), and 51.7% (15/29 cycles), respectively, with a clinical pregnancy at the first ET (*p* < .001), and cumulative ongoing pregnancy rates of 85.2% (23/27 women), 30.8% (4/13 women), and 78.3% (18/23 women), respectively, in two ET cycles (*p* = .002; Table 4). Despite having received treatment for CE, patients in the CE group had a poor pregnancy outcome.

**Table 3:** Ratio of recovery from chronic endometritis

	CE group	Cured‐CE group
	*n* = 19	*n* = 36
Recovery rate from CE^a^, *n* (%)		
First treatment with antibiotics	7/19 (36.8)	29/36 (80.6)
Second treatment with antibiotics	10/12 (83.3)	7/7 (100)
Third treatment with D&C or antibiotics	2/2 (100)	‐
Total recovery rate	19/19 (100)	36/36 (100)

Abbreviation: CE, chronic endometritis; ERA, endometrial receptivity array.

^a^ CE were treated prior or posterior to the ERA test in cured‐CE or CE groups, respectively.

**Table 4:** Pregnancy outcomes after personalized embryo transfer

	**Non‐CE group**	**CE group**	**Cured‐CE group**	
	***n*** **=** **27**	***n*** **=** **18**	***n*** **=** **29**	***p* Value**
First personalized ET				
No of transferred embryos, mean ± *SD*	1.1 ± 0.3	1.1 ± 0.2	1.0 ± 0.2	.512
ET with morphologically good blastocysts, *n* (%)	23 (85.2)	17 (94.4)	25 (86.2)	.736
Pregnancy outcomes, *n* (%)				
hCG positive rate	23 (85.2)	6 (33.3)	17 (58.6)	**.002**
Clinical pregnancy rate	21 (77.8)	4 (22.2)	15 (51.7)	**<.001**
Miscarriage rate	3 (14.3)	1 (25.0)	4 (26.7)	.521
Ongoing pregnancy rate	18 (66.7)	3 (16.7)	11 (37.9)	**.003**

	***n* = 9**	***n* = 10**	***n* = 12**	***p* Value**
Second personalized ET				
No of transferred embryos, mean ± *SD*	1.1 ± 0.3	1.0 ± 0	1.3 ± 0.5	.102
ET with morphologically good embryos^a^, *n* (%)	8 (88.9)	9 (90.0)	9 (75.0)	.703
Pregnancy outcomes, *n* (%)				
hCG positive rate	6 (66.7)	3 (30.0)	9 (75.0)	.126
Clinical pregnancy rate	6 (66.7)	2 (20.0)	8 (66.7)	.057
Miscarriage rate	1 (20.0)	1 (50.0)	1 (12.5)	.486
Ongoing pregnancy rate	5 (55.6)	1 (10.0)	7 (58.3)	.053
Cumulative ongoing pregnancy rate in 2 ET cycles, *n* (%)	***n*** **=** **27**	***n*** **=** **13**	***n*** **=** **23**	
	23 (85.2)	4 (30.8)	18 (78.3)	**.002**

Abbreviations: CE, chronic endometritis; ET, embryo transfer.

^a^ Morphologically good embryos were diagnosed as 5‐ or 6‐day blastocysts after fertilization, except for grade C in both the inner cell mass and the trophectoderm of the Gardner classification.

## 4 DISCUSSION

Endometrial stromal cells from women with CE show impaired secretion and lower messenger RNA expression of the decidual markers prolactin (*prolactin*) and insulin‐like growth factor binding protein‐1 in vitro compared with those from women without CE.^30^ This suggests that CE inhibits optimal decidual transformation of the endometrium and shifts or disappears the WOI, leading to acquired implantation failure. In our study, patients in the CE group had a significantly lower rate of receptive endometria compared with the non‐CE and cured‐CE groups according to the results of ERA testing. Therefore, CE might inhibit decidualization of endometrium and adversely affect the WOI in vivo as it has been shown to do in primary cultures in vitro. To the best of our knowledge, this is the first report to describe the impact of CE on ERA test results.

Human endometrium transiently acquires a specific phenotype for receiving a competent embryo at implantation.^1^ Impaired decidual transformation leading to an endometrium with a non‐receptive state is one cause of infertility and pregnancy loss, and is the rate‐determining step in IVF treatment.^1^, ^36^, ^37^ The ERA is a novel test for objectively identifying the timing for an individual's WOI. This test is based on the assumption that the individual WOI is constant, but this has not yet been proven. Impaired decidualization caused by CE is expected to delay the WOI; however, 62.5% (10/16 cases) of the women with CE in our study had personalized WOIs of P + 4 or P + 4.5, meaning that the implantation period might be brought forward by the CE. Embryo implantation is associated with a transient inflammatory reaction^38^, ^39^, ^40^, ^41^; therefore, biopsies of endometria in patients with CE might be diagnosed by ERA testing as an earlier WOI rather than an optimal WOI based on the expression of secreted proinflammatory‐related genes.

The ERA results and pregnancy outcomes in patients who were treated and recovered from CE were similar to those who had never been diagnosed with CE (the non‐CE group). A WOI that is impaired by CE may be reversible with appropriate therapies. Previous studies also showed that pregnancy and live birth rates in women after recovery from CE were significantly higher than those with active CE.^25^, ^26^, ^27^ In fact, a receptive endometrium was confirmed by endometrial biopsy after treatment for CE in two women out of three in the CE group (Figure S1). Therefore, diagnosis and treatment of CE should be done before performing the ERA test. Furthermore, ERA results in patients with CE was unreliable based on the poor pregnancy outcome after personalized ET in our study, despite successful recovery from CE. The results of ERA obtained in the presence of CE gets different after CE is cured. Because the therapeutic efficacy of ERA testing for patients with a history of RIF remains uncertain,^16^, ^17^, ^18^, ^19^ we propose that, when CE is found in patients with RIF, they should be treated before performing additional ERA tests. Moreover, the necessity of ERA test in patients with CE should be further investigated, because ERA tests are expensive and most of the patients can become pregnant after recovery from CE.

This study has some limitations. The patients had undergone endometrial samplings for the ERA test and IHC for CD138 within 3 months, but they were not the same biopsy. Additionally, there are still no global standard diagnostic criteria for CE. Therefore, we defined ≥5 CD138‐positive cells per 10 nonoverlapping random stromal areas as CE; however, it should be noted that different diagnostic criteria may yield different results. With regard to pregnancy outcomes, some of the data for patients who did not undergo ET in our clinic were lost to follow‐up.

In conclusion, CE modified the individual WOI, leading to embryo–endometrial asynchrony; therefore, it is not recommended to transfer embryos based on the WOI obtained by performing ERA in the presence of CE. Diagnosis and treatment of CE should be done before ERA testing. Prospective controlled trials are required to confirm the impact of CE on ERA results and pregnancy outcomes following personalized ET.

## CONFLICT OF INTERESTS

The authors declare that there are no conflict of interests.

## AUTHOR CONTRIBUTIONS

Conceptualization, formal analysis, investigation, methodology, resources, supervision, visualization, and writing: Keiji Kuroda. Collecting resources and discussion: all authors. Project administration: Rikikazu Sugiyama.

## Supporting information

Supporting information.

Supporting information.

## Data Availability

The data that support the findings of this study are available on request from the corresponding author. The data are not publicly available due to privacy or ethical restrictions.
