# Supplementary figures and images for: Impact of chronic endometritis on endometrial receptivity analysis results and pregnancy outcomes
Source: Immun Inflamm Dis. 2020 Sep 23;8(4):650–8. doi: 10.1002/iid3.354 (PMC7654412; doi:10.1002/iid3.354)

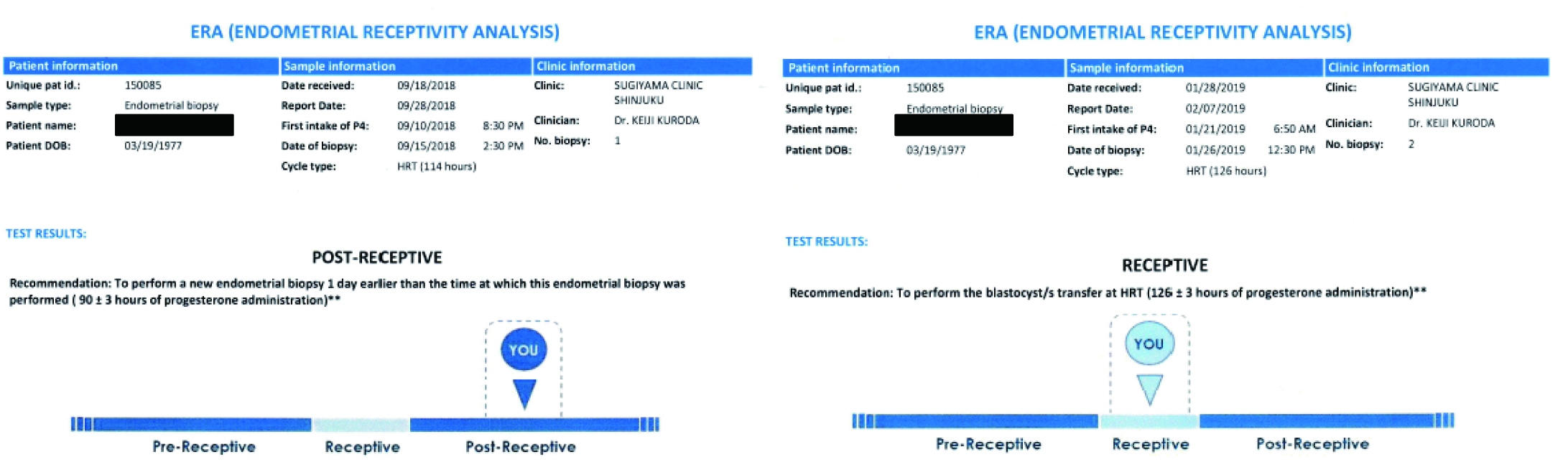

Supplement: Supplementary file 1 — Supporting information. [file IID3-8-650-s001.tif]
