# Supplementary material for: Impact of chronic endometritis on endometrial receptivity analysis results and pregnancy outcomes
Source: Immun Inflamm Dis. 2020 Sep 23;8(4):650–8. doi: 10.1002/iid3.354 (PMC7654412; doi:10.1002/iid3.354)
Supplement: Supplementary file 2 — Supporting information. [file IID3-8-650-s002.docx]

**Supplemental figure 1. Case: A 42-year-old woman with a history of implantation failure after 4 embryo transfer cycles in previous hospital**

First biopsy showed 98 CD138-positive cells in 10 nonoverlapping random stromal areas at 400-fold magnification, no bacterium in intrauterine bacterial culture, and post-receptive with 90±3h of recommendation timing of embryo transfer in ERA test, therefore chronic endometritis (CE) was diagnosed. Doxycycline, 100 mg twice daily for 2 weeks were administered.

Second biopsy demonstrated 30 CD138-positive cells, no bacterium in intrauterine bacterial culture; thus, a combination of amoxicillin, azithromycin, metronidazole, and antibiotics-resistant lactic acid bacteria, twice a day for 2 weeks twice daily for 2 weeks were administered.

In third biopsy, CE was cured with 1 CD138-positive cell, but *Candida glabrata* was detected in intrauterine bacterial culture; thus, fluconazole, 100 mg twice daily for 2 weeks were administered.

As forth biopsy, ERA test showed receptive with 126±3h of recommendation timing of embryo transfer.
